# Caveolin-3 differentially orchestrates cholinergic and serotonergic constriction of murine airways

**DOI:** 10.1038/s41598-018-25445-1

**Published:** 2018-05-14

**Authors:** M. Keshavarz, M. Skill, M. I. Hollenhorst, S. Maxeiner, M. Walecki, U. Pfeil, W. Kummer, G. Krasteva-Christ

**Affiliations:** 1 Institute of Anatomy and Cell Biology, Justus-Liebig-University Giessen, Giessen, Germany; 2 German Center for Lung Research (DZL), Marburg, Germany; 3 Institute of Anatomy and Cell Biology, School of Medicine, Saarland University, Saarbrucken, Germany; 4 Present Address: Institute of Anatomy and Cell Biology, School of Medicine, Saarland University, Saarbrucken, Germany

## Abstract

The mechanisms of controlling airway smooth muscle (ASM) tone are of utmost clinical importance as inappropriate constriction is a hallmark in asthma and chronic obstructive pulmonary disease. Receptors for acetylcholine and serotonin, two relevant mediators in this context, appear to be incorporated in specialized, cholesterol-rich domains of the plasma membrane, termed caveolae due to their invaginated shape. The structural protein caveolin-1 partly accounts for anchoring of these receptors. We here determined the role of the other major caveolar protein, caveolin-3 (cav-3), in orchestrating cholinergic and serotonergic ASM responses, utilizing newly generated cav-3 deficient mice. Cav-3 deficiency fully abrogated serotonin-induced constriction of extrapulmonary airways in organ baths while leaving intrapulmonary airways unaffected, as assessed in precision cut lung slices. The selective expression of cav-3 in tracheal, but not intrapulmonary bronchial epithelial cells, revealed by immunohistochemistry, might explain the differential effects of cav-3 deficiency on serotonergic ASM constriction. The cholinergic response of extrapulmonary airways was not altered, whereas a considerable increase was observed in cav-3^−/−^ intrapulmonary bronchi. Thus, cav-3 differentially organizes serotonergic and cholinergic signaling in ASM through mechanisms that are specific for airways of certain caliber and anatomical position. This may allow for selective and site-specific intervention in hyperreactive states.

## Introduction

The mechanisms of controlling airway smooth muscle (ASM) tone are of utmost clinical importance since excessive sensitivity to contractile stimuli, called bronchial hyperresponsiveness (BHR), is considered as a hallmark in asthma and chronic obstructive pulmonary disease COPD^1,2^. Acetylcholine (ACh), released from parasympathetic nerve fibres, is the dominant constrictory neurotransmitter in the airways, acting via muscarinic ACh receptors (mAChR) types 2 and 3^3^. In animal models of BHR, the release of ACh from nerve terminals is considerably increased^4,5^. During inflammation, a wide range of additional mediators is released acting either directly on bronchial smooth muscle cells (SMC) or indirectly through neural pathways leading to BHR^5^. Among them is serotonin (5-hydroxytryptamine, 5-HT), a secretory product of mast cells, affecting ASM tone *in situ* and *in vitro*^6–8^. It has been shown that increased levels of free 5-HT are present in the plasma of symptomatic asthmatic patients compared to asymptomatic patients^9,10^. The subtypes of 5-HT receptors present in airway SMC and serotonergic effects appear to be species-dependent^11^. We recently showed the expression of 5-HT1B, 5-HT2A, 5-HT6 and 5-HT7 receptors as the most prevalent subunits in the airways of C57BL/6 J mice^12^. In humans, 5-HT1A receptors are responsible for bronchodilation and bronchoconstriction is attributed to 5-HT2A receptors on ASM^9^.

It was previously suggested that both, serotonergic and cholinergic signaling in ASM are orchestrated by specialized plasma membrane domains termed caveolae^13^. These are cholesterol-rich, flask-shaped membrane invaginations that concentrate numerous receptor kinases, structural proteins, G-protein-coupled receptors (GPCR) and ion channels. They are important in pathways associated with calcium homeostasis, migration, proliferation of cells, mechanosensation and ASM constriction^13–21^. The principal structural proteins of caveolae are caveolins (cav). Three isoforms are known: cav-1 and cav-3 are essential for caveolae formation and serve as binding partners for receptors and enzymes, whereas cav-2 is an auxiliary isoform that is generally coexpressed with cav-1^16^. Expression of cav isoforms varies greatly from tissue to tissue^22^. Cav-1 is widely expressed in endothelial cells, type I pneumocytes, fibroblasts, adipocytes, and SMC^16,23^. Cav-3 is highly expressed in striated (skeletal and cardiac) muscle and certain SMC and is critical for caveolae formation in the absence of cav-1^23–25^. Cav-1 and cav-3 can also be coexpressed, cav-1/cav-3 hetero-oligomeric complexes were also observed in rat and mouse myocytes from mice overexpressing cav-1^26,27^.

General disruption of cholesterol-rich microdomains and genetic ablation of cav-1 result in specific functional impairments along the airway tree. Cholesterol depletion with methyl-β-cyclodextrin (MCD) and cav-1 deficiency abolished the constrictor response to 5-HT in murine trachea and extrapulmonary airways^12,13^. Likewise, cholesterol depletion impaired serotonergic responses of bovine tracheal SMC^28^. Serotonergic constriction of murine intrapulmonary airways, however, remained unchanged in cav-1 deficient mice, as assessed by videomicroscopic analysis of precision cut lung slices (PCLS)^12^. Cholinergic constriction is differentially affected by MCD treatment and cav-1 deficiency, although both interventions reduce muscarinic calcium mobilization in airway SMC^20^. We previously observed an about 50% decrease in muscarinic bronchonconstriction in MCD-treated PCLS while the muscarinic response was unaffected in intrapulmonary bronchi from cav-1^−/−^ mice^12^, consistent with an undisturbed cholinergic tracheal constriction in these mice^29^.

These data imply that additional constituents of cholesterol-rich microdomains, rather than cav-1 alone, are essential for receptor-mediated ASM constriction, with varying contributions depending on airway caliber and anatomical position. As we have shown previously that both cav-1 and cav-3 are present and interact in murine ASM^13^, cav-3 appears as a candidate for serving this function. Previous studies addressing cav-3 interaction with serotonergic and cholinergic signaling have primarily focused upon cardiac muscle cells. There, 5-HT2A receptors interact with cav-3 upon stimulation with 5-HT, and cav-3 silencing enhanced the myocyte hypertrophic response^30^. Similarly, agonist stimulation triggers association of cav-3 with the dominant cholinergic receptor of cardiomyocytes, M2R, and both can then be co-purified with the endothelial nitric oxide synthase isoform (eNOS) from plasmalemmal fractions^31^. Such eNOS/cav-3 interaction is assumed to hold a key role in cholinergic modulation of cardiac myocyte function^32^.

In contrast, the functional role of cav-3 in SMC is poorly defined. In the female rat urinary bladder, protein levels of cav-3 were significantly lower after ovariectomy and restored to control levels after 17β-estradiol treatment which correlated to changes in detrusor muscle overactivity, but a causal relationship remains to be investigated^33^. In vascular smooth muscle, cav-3 has been associated with maintaining or promoting a switch to the contractile phenotype^34^. Functional studies directly addressing its role in ASM are lacking. On this background we aimed to assess the detailed distribution of cav-3 protein and its functional role in cholinergic and serotonergic airway constriction. To this end, immunohistochemical localization and co-immunoprecipitation studies were conducted, and cav-3 deficient mice were generated for functional analyses. Given the identified regional differences in airway constrictor responsiveness and underlying mechanisms along the airway tree^12,35^, murine tracheal segments (cranial, middle and caudal), extra- and intrapulmonary bronchi were separately investigated in organ bath experiments (extrapulmonary airways) and by videomorphometry in PCLS.

## Results

### Cav-3 and caveolae in tracheal and bronchial SM

**Figure 1:**
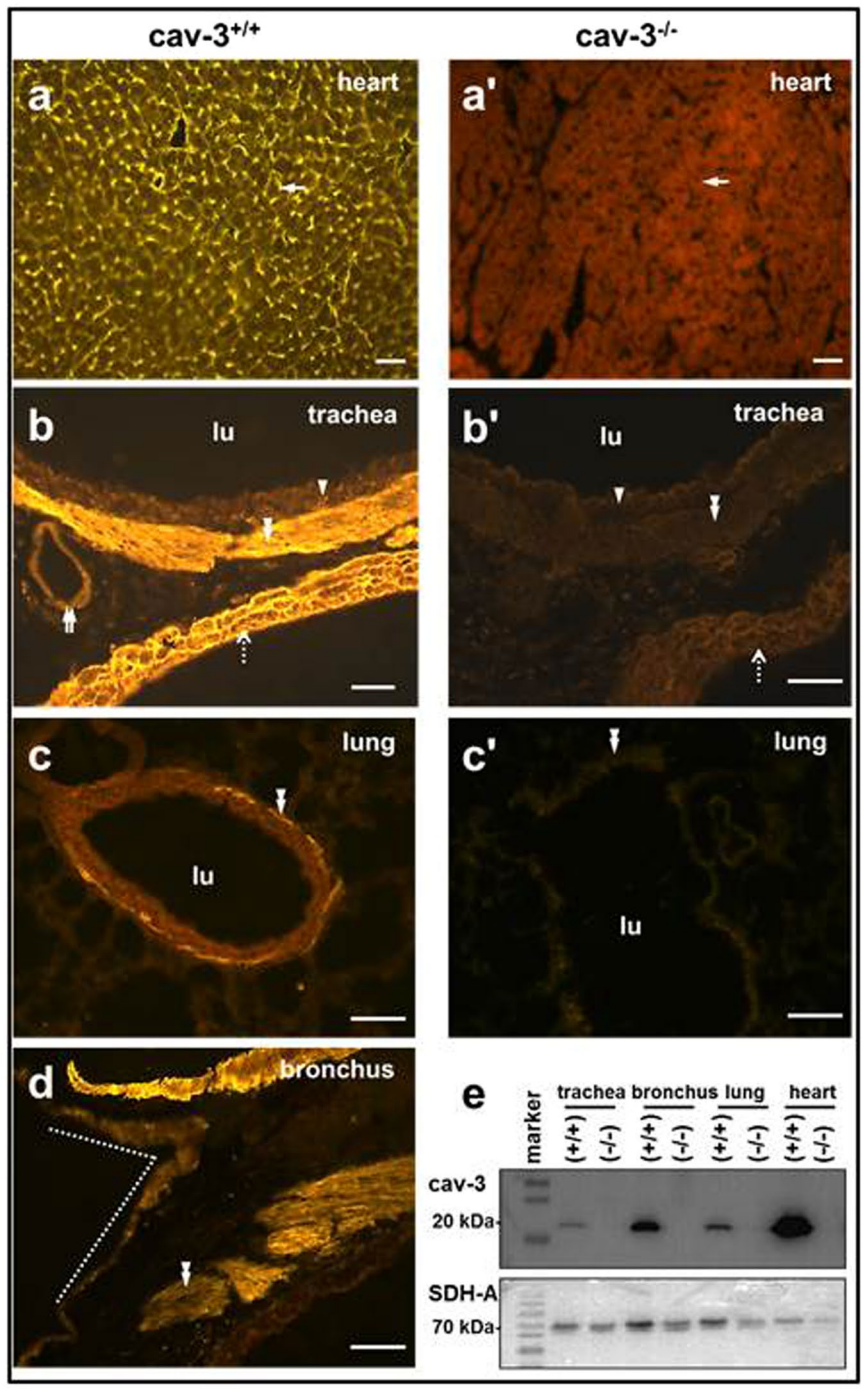
Immunofluorescence labelling for cav-3 in cav-3^+/+^ and cav-3^−/−^ mice. Representative images for cav-3^+/+^ (*n* = 4 animals) and cav-3^−/−^ mice (*n* = 5 animals). (**a**–**d**) Cav-3 immunoreactivity is seen in the cardiac muscle cells (arrow), tracheal epithelium (arrowhead), tracheal and bronchial SMC (double arrowhead), and cardiomyocytes of the pulmonary vein (dashed arrow) and arterial smooth muscle (double arrow) in cav-3^+/+^ mice. (**d**) SMC are shown in the left extrapulmonary bronchus and the tracheal bifurcation is also shown by a dotted line. No cav-3 labelling is noted in cav-3^−/−^ mice. Bar = 50 µm. (**e**) Specificity control of the anti-cav-3 antibody. No cav-3 immunolabelling is present in cav-3^−/−^ mouse samples, while the antibody recognizes a single 20 kDa protein band in protein extracts from cav-3^+/+^ mouse samples. Our own laboratory polyclonal antibody to succinate dehydrogenase complex, subunit A (SDH-A) labels a protein band at 70 kDa in all cav-3^−/−^ and cav-3^+/+^ mouse samples as a reference control.

Cav-3-immunolabelling was demonstrated in cardiac muscle cells in the heart that served as a positive control tissue (Fig. 1a). The same cav-3-antibody labeled tracheal, intra- and extrapulmonary bronchial SMC (Fig. 1b–d). Cav-3 immunolabelling was observed in the tracheal epithelium (Fig. 1b). No cav-3-labelling was noted in the bronchial epithelium (Fig. 1c). The specificity of the cav-3 antibody has been established by the absence of cav-3-immunolabelling in cav-3^−/−^ mice (Fig. 1a’–c’). Western blotting supported the immunohistochemical findings since the cav-3-antibody recognized a ≈20 kDa band in protein extracts from different tissues only in cav-3^+/+^ mice while it did not label the band in protein extracts from cav-3^−/−^ mice (Fig. 1e).

**Figure 2:**
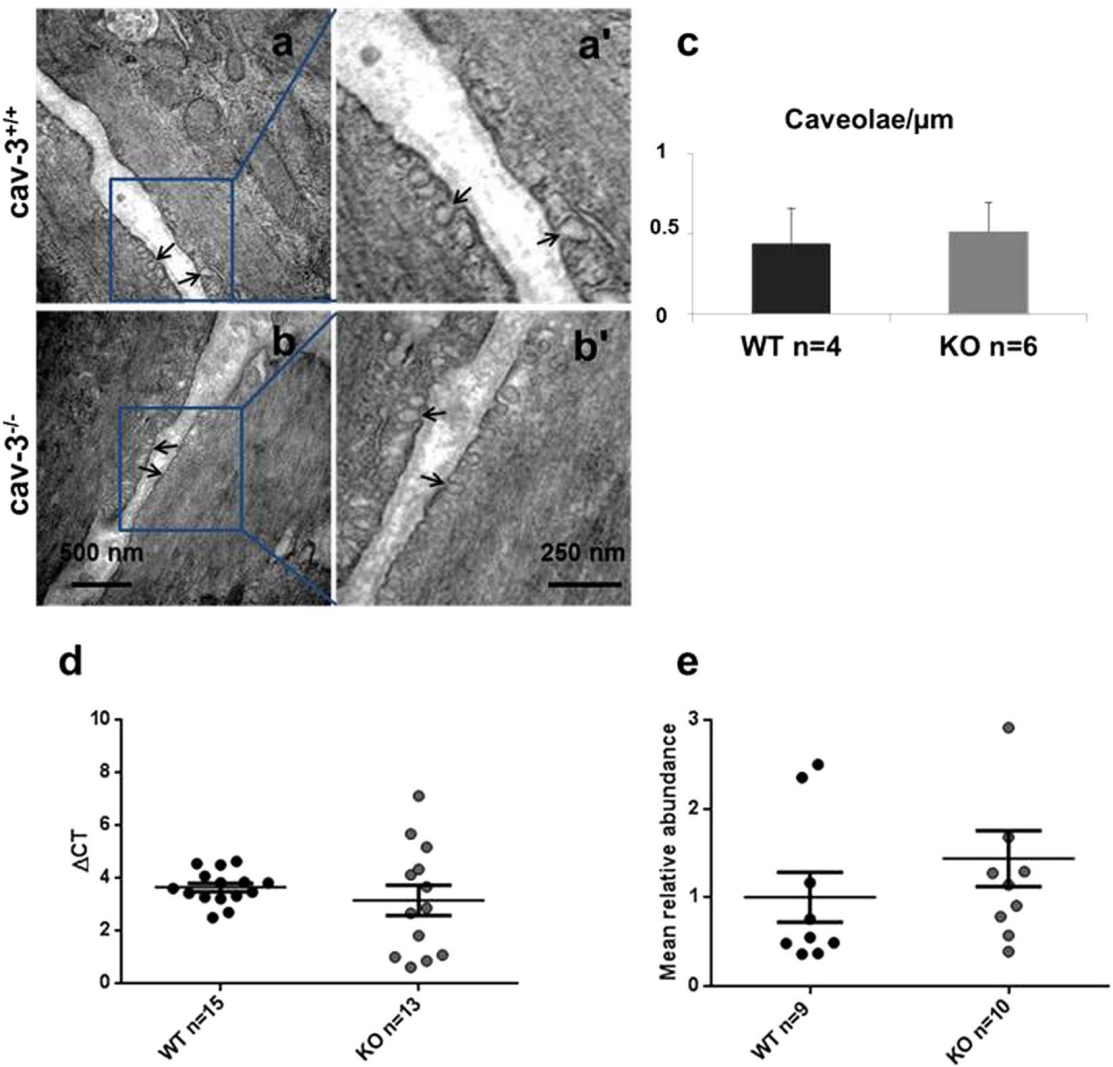
Ultrastructure of caveolae in airway smooth muscle from cav-3^+/+^ and cav-3^−/−^ mice. Representative images for tracheae of cav-3^+/+^ mice (WT, n = 4 animals) and cav-3^−/−^ (KO, n = 6 animals). (**a**-a’) Cav-3^+/+^ mice. Numerous caveolae are located in groups at the plasma membrane of tracheal smooth muscle cells (SMC). (**b**-b’) Cav-3^−/−^ mice. Tracheal SMC have the same number of caveolae. Caveolae = arrowhead. (**c**) Quantification of caveolae in the tracheal SMC from cav-3^+/+^ and cav-3^−/−^ mice. Multiple electron micrographs were obtained for each tissue, and both the number of caveolae and total length of plasma membrane present were quantified in each image. Caveolae were counted as omega-shaped membrane profiles open at the cell surface. (**d**) Real-time PCR for EHD-2 in cav-3^+/+^ and cav-3^−/−^ mouse tracheae, relative expression is presented as ΔCT compared to β-actin. Lower ΔCT reflects higher expression. (**e**) Densitometry from EHD2 western blots compared to β-tubulin, to allow distribution of EHD2 protein to be compared in cav-3^+/+^ and cav-3^−/−^ mouse tracheae. Bars represent SEM, values are means from separate experiments (n). P-values were calculated using an Student’s unpaired t-test.

The impact of cav-3 depletion for the maintenance of caveolar structure in tracheal SM was assessed by electron microscopy. Neither the number of caveolae (per μm of the plasma membrane) nor their ultrastructural appearances were altered in cav-3^−/−^ mice (Fig. 2a–c). Additionally, mRNA and protein of EHD2, a caveolae abundance marker, were consistently expressed in samples from cav-3^+/+^ and cav-3^−/−^ mice (Fig. 2d,e).

**Figure 3:**
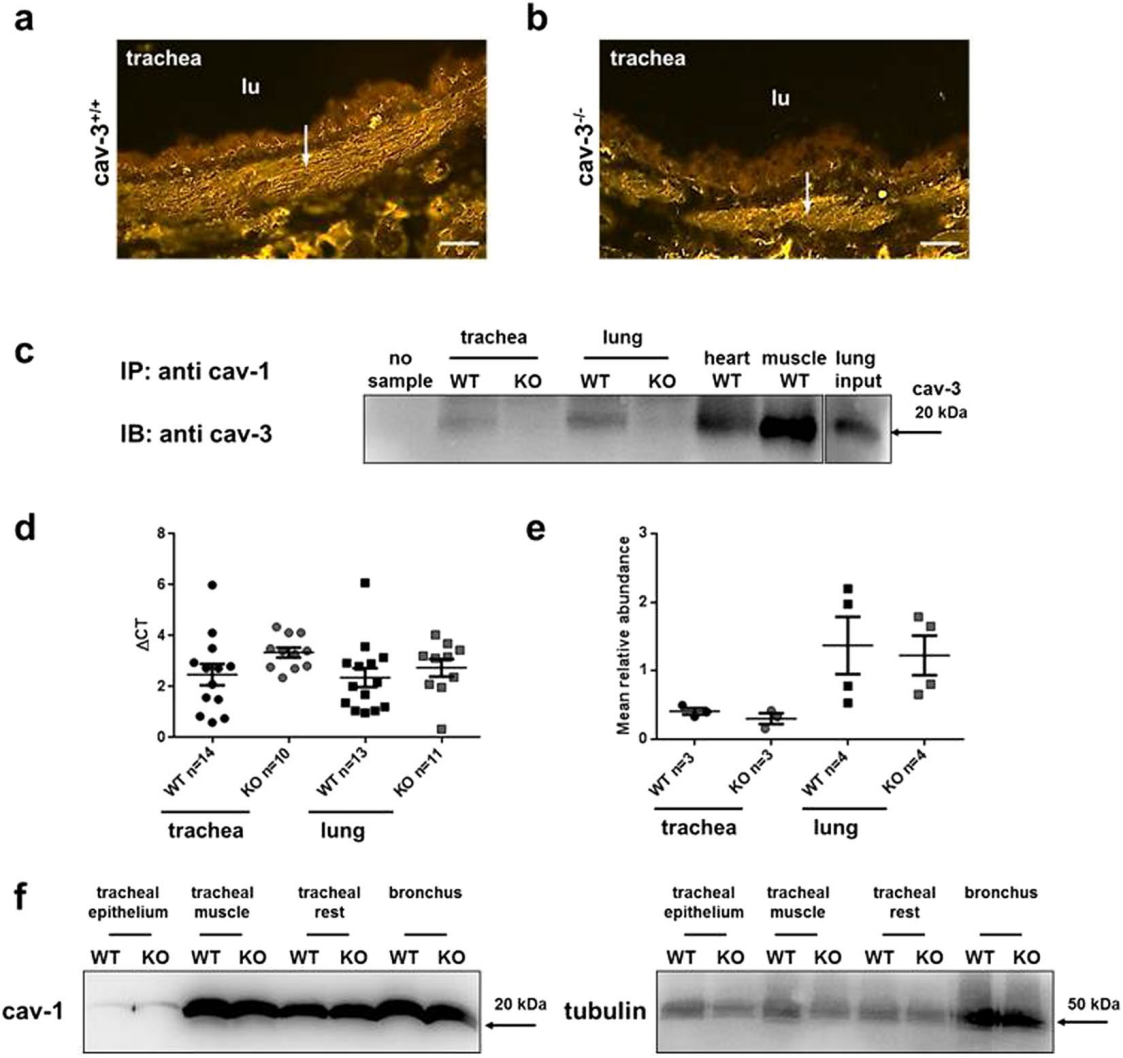
Cav-1 expression in cav-3^+/+^ (WT) and cav-3^−/−^ (KO) mice. (**a**,**b**) Tracheal SMC are cav-1-immunoreactive (arrow) in both mouse strains. Bar = 50 µm. (**c**) Immunoprecipitates (IP) with cav-1 are immunoblotted (IB) for cav-3. Positive Co-IP controls include heart and skeletal muscle from cav-3^+/+^ mice. Lung input as intact protein lysate from cav-3^+/+^ mice is blotted and the expression level of cav-3 protein is observed with longer exposure time. The negative control of Co-IP includes beads and antibody in the absence of lysate (no sample). (**d**) Real-time PCR for cav-1 in cav-3^+/+^ and cav-3^−/−^ mice trachea and lung homogenates, relative expression is presented as ΔCT compared to β-actin. Lower ΔCT reflects higher expression. Cav-1 was significantly different in tracheal muscle of cav-3^−/−^ and cav-3^+/+^ mice. (**e**) Densitometry from cav-1 Western blots, to allow distribution of cav-1 protein compared to β-tubulin to be compared in cav-3^+/+^ and cav-3^−/−^ mouse trachea and lung. Bars represent SEM. Values are means from separate experiments (n). Each individual experiment was analyzed by the Mann-Whitney U-test. *p ≤ 0.05. (**f**) Cav-1 staining is strong in tracheal muscle, bronchus and trachea without epithelium and smooth muscle (tracheal rest), while being faint in tracheal epithelium. Tubulin-immunolabelling is observed in the preparations from cav-3^+/+^ and cav-3^−/−^ mice.

Similar patterns of immunolabelling for cav-1 were observed in tracheal muscle in cav-3^−/−^ and cav-3^+/+^ mouse strains (Fig. 3a,b). In addition, co-immunoprecipitation (Co-IP) revealed that cav-1 and cav-3 interact in trachea and lung tissues (Figs 3c, S3). Although, the cav-1 mRNA expression was higher in tracheal muscle of cav-3^+/+^ mice than in cav-3^−/−^ mice, Western blotting analyses showed that the amount of cav-1 protein was not changed in both, cav-3^−/−^ and cav-3^+/+^ mouse strains (Fig. 3d,e). These findings demonstrate that cav-3 is not involved in the regulation of the tracheal cav-1 protein abundance.

### Muscarine-induced contraction of extrapulmonary airways

**Figure 4:**
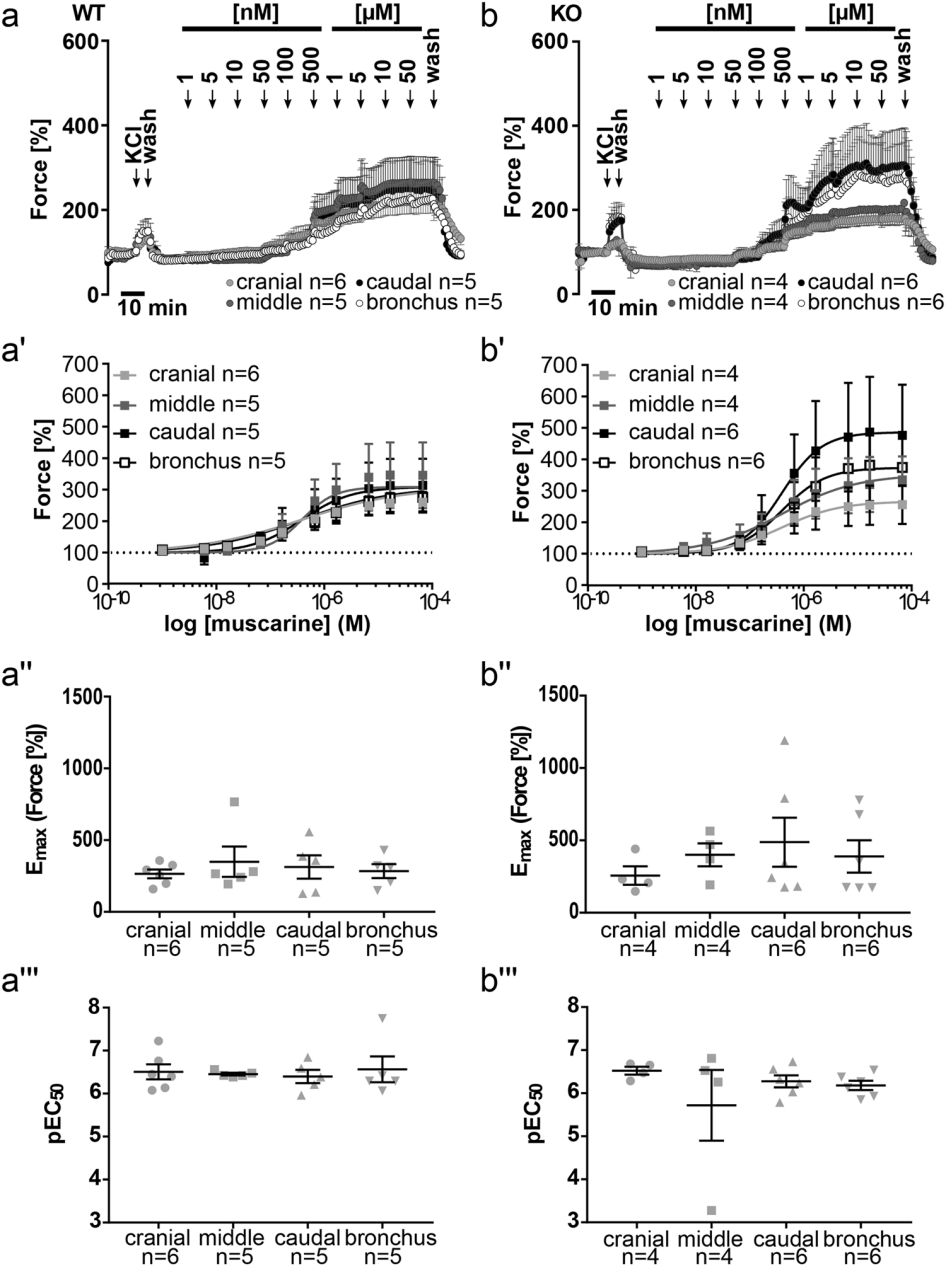
Changes in constrictor force and reactivity in response to muscarine in various airway segments from cav-3^+/+^ (WT) and cav-3^−/−^ (KO) mice. Tension changes were measured in force [grams]. After equilibration, baseline tension was always accustomed to 0.5 g. Baseline was set as 100% and the maximum response at each concentration was evaluated. Each point represents the mean number of animals (n) ± SEM. (**a**,**b**) Concentration-dependent contraction of ASM was induced by muscarine in cav-3^+/+^ (**a**) and cav-3^−/−^ (**b**) mouse strains. All sigmoidal concentration-response curves shown here were plotted according to the Hill equation. (a’-b’) Depicted is the constrictor force of different tracheal parts and extrapulmonary bronchi in cav-3^+/+^ (a’) and cav-3^−/−^ (b’) mice. No difference in maximal responses (E_max_, a” and b”) and pEC_50_ (a”’ and b”’) values was observed in cav3^+/+^ and cav3^−/−^ mice between tracheal parts or bronchi when data were analyzed by One-Way ANOVA and subjected to Dunnett’s multiple comparisons test afterwards.

**Figure 5:**
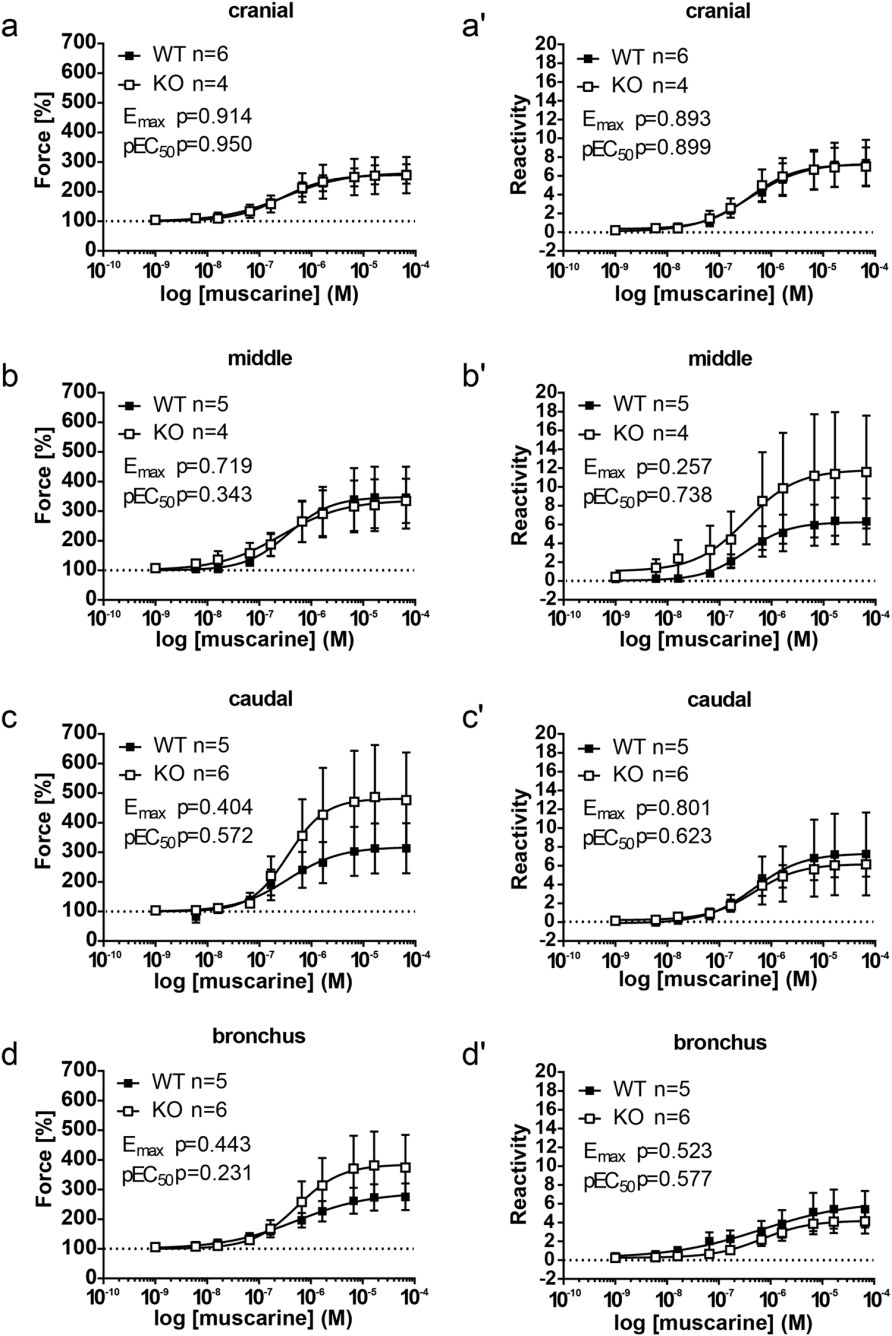
Comparison of force and reactivity in response to muscarine between cav-3^+/+^ (WT) and cav-3^−/−^ (KO) mice. For each individual experiment the maximal responses (E_max_) and pEC_50_ values for muscarine in force level (**a**–**d**) and airway reactivity (a’–d’) were estimated. All sigmoidal concentration-response curves were plotted according to the Hill equation. (a’–d’) The airway reactivity represents the constrictor response to muscarine compared to the corresponding KCl response. All data were analyzed with the Student’s unpaired t-test. Each point is shown as the mean number of animals (n) ± SEM. There is no difference between the cav-3^−/−^ and the cav-3^+/+^ airway segments with regards to reactivity level and contraction force.

All tracheal segments as well as extrapulmonary bronchi exhibited a concentration-dependent muscarinic contraction in cav-3^+/+^ and cav-3^−/−^ mouse strains (Fig. 4a,b). The pEC_50_ and constrictor potency values were not significantly different between the tracheal parts and extrapulmonary bronchi of cav-3^+/+^ mice (cranial force: E_max_ = 263.82 ± 31.16, pEC_50_ = 6.51 ± 0.17; middle force: E_max_ = 348.44 ± 105.40, pEC_50_ = 6.45 ± 0.03; caudal force: E_max_ = 312.16 ± 81.18, pEC_50_ = 6.40 ± 0.15; bronchus force: E_max_ = 283.66 ± 48.72, pEC_50_ = 6.56 ± 0.30) (Fig. 4a’-a’’’) and cav-3^−/−^ mice (cranial force: E_max_ = 256.70 ± 63.76, pEC_50_ = 6.52 ± 0.09; middle force: E_max_ = 400.15 ± 79.43, pEC_50_ = 5.72 ± 0.82; caudal force: E_max_ = 487.75 ± 169.03, pEC_50_ = 6.28 ± 0.14; bronchus force: E_max_ = 389.02 ± 111.96, pEC_50_ = 6.18 ± 0.11) (Fig. 4b’-b’’’). Neither in absolute force (Fig. 5a–d) nor in reactivity related to KCl-induced contraction (Fig. 5a’–d’), a significant difference between airways taken from cav-3^−/−^ and cav-3^+/+^ mice was detected. In both strains, the maximum effect evoked by muscarine and pEC_50_ were not significantly different for the individual tracheal segments and extrapulmonary bronchi.

### 5-HT-induced contraction of extrapulmonary airway

**Figure 6:**
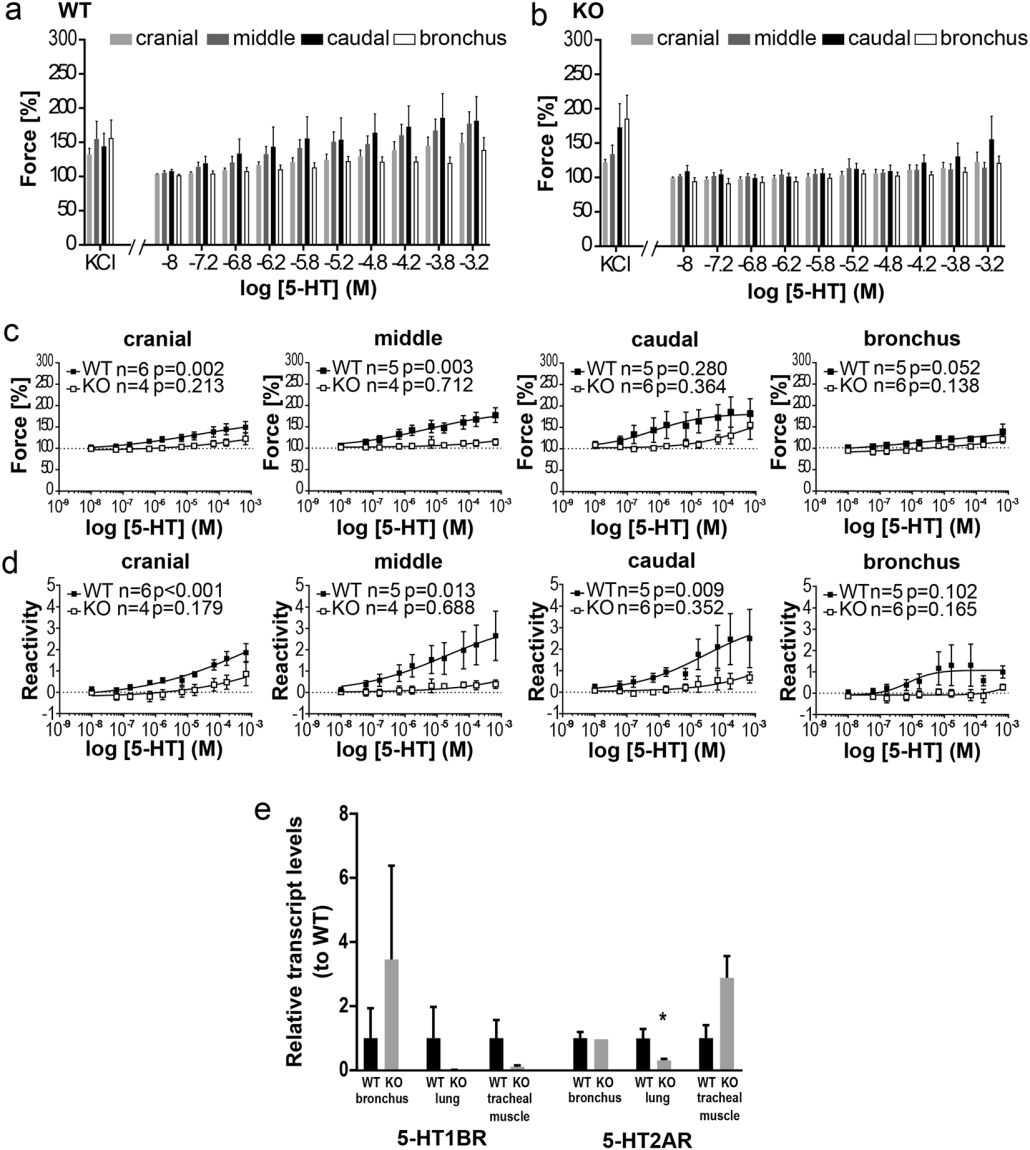
Changes in constrictor force and reactivity of various extrapulmonary airway segments from cav-3^+/+^ (WT) and cav-3^−/−^ (KO) mice induced by 5-HT. In the organ bath recordings, each point represents the mean number of animals (n) ± SEM. After equilibration, baseline tension was adjusted to 0.5 g. Baseline was set as 100% and the maximum response at each concentration was calculated. (**a**,**b**) Changes in constrictor force after additive application of 5-HT. 5-HT induces concentration-dependent contraction in the cranial and middle part of trachea from cav-3^+/+^ mice, whereas the trachea of cav-3^−/−^ mice and the extrapulmonary bronchi are not responsive to 5-HT. (**c**,**d**) Comparison of constrictor response (force and reactivity) between both mouse strains. Cav-3^+/+^ mice showed significant constriction in the cranial and middle part of the trachea, whereas there was no significant constriction in the caudal part and the extrapulmonary bronchi. In contrast to this neither the tracheal segments nor the extrapulmonary bronchi constricted in cav3^−/−^ mice. All tracheal segments of cav3^+/+^ mice showed a significant reactivity, whereas the reactivity of the extrapulmonary bronchi was not significant. Reactivity was abolished in cav-3^−/−^ mice. Data were analyzed with One-Way ANOVA or the Kruskal-Wallis test followed by Dunnett’s test for multiple comparisons, depending on normal distribution established with the Shapiro-Wilk normality test. (**e**) Relative transcript levels of 5-HT1BR and 5-HT2AR genes standardized on internal β-microglobulin (β-MG) levels. Results for the cav-3^−/−^ tissues are presented relative to the results for cav-3^+/+^ tissues set to 1 to appreciate its potential differences independent from individual assay performance. Data are presented as mean ± SEM (Student’s t-test, *p < 0.05; n = 4 mice per genotype).

In cav-3^+/+^ mice, the amplitude in the force of the 5-HT-induced contraction was concentration-dependent in the cranial and middle tracheal segments whereas contraction in the caudal segment and extrapulmonary bronchi was not statistically significant (Fig. 6a,c). An increase in reactivity could be observed in all tracheal segments of cav-3^+/+^ mice, but not in extrapulmonary bronchi (Fig. 6d). Interestingly, the reactivity response to cumulative application of 5-HT and the force were eliminated in all tracheal segments and extrapulmonary bronchi of cav-3^−/−^ mice (Fig. 6b–d). The reaction to 60 mM KCl was preserved at the end of experiments in cav-3^−/−^ mice indicating the viability of the preparations. No significant changes in expression of 5-HT1BR and 5-HT2AR were observed in tracheal SM and in extrapulmonary bronchi in cav-3^+/+^ and cav-3^−/−^ mice (Fig. 6e). Expression of 5-HT2AR was significantly reduced only in the lungs of cav-3^−/−^ mice. 5-HT1AR-, 5-HT6R- and 5-HT7R-expression were inconsistently detected in the airways and lungs from cav-3^+/+^ and cav-3^−/−^ mice. Since the 5-HTR expression was comparable in tracheal and bronchial ASM in cav-3^+/+^ and cav-3^−/−^ mice, the differences in the constrictor response cannot be explained with transcriptional changes.

### Muscarine-induced contraction of intrapulmonary airways

**Figure 7:**
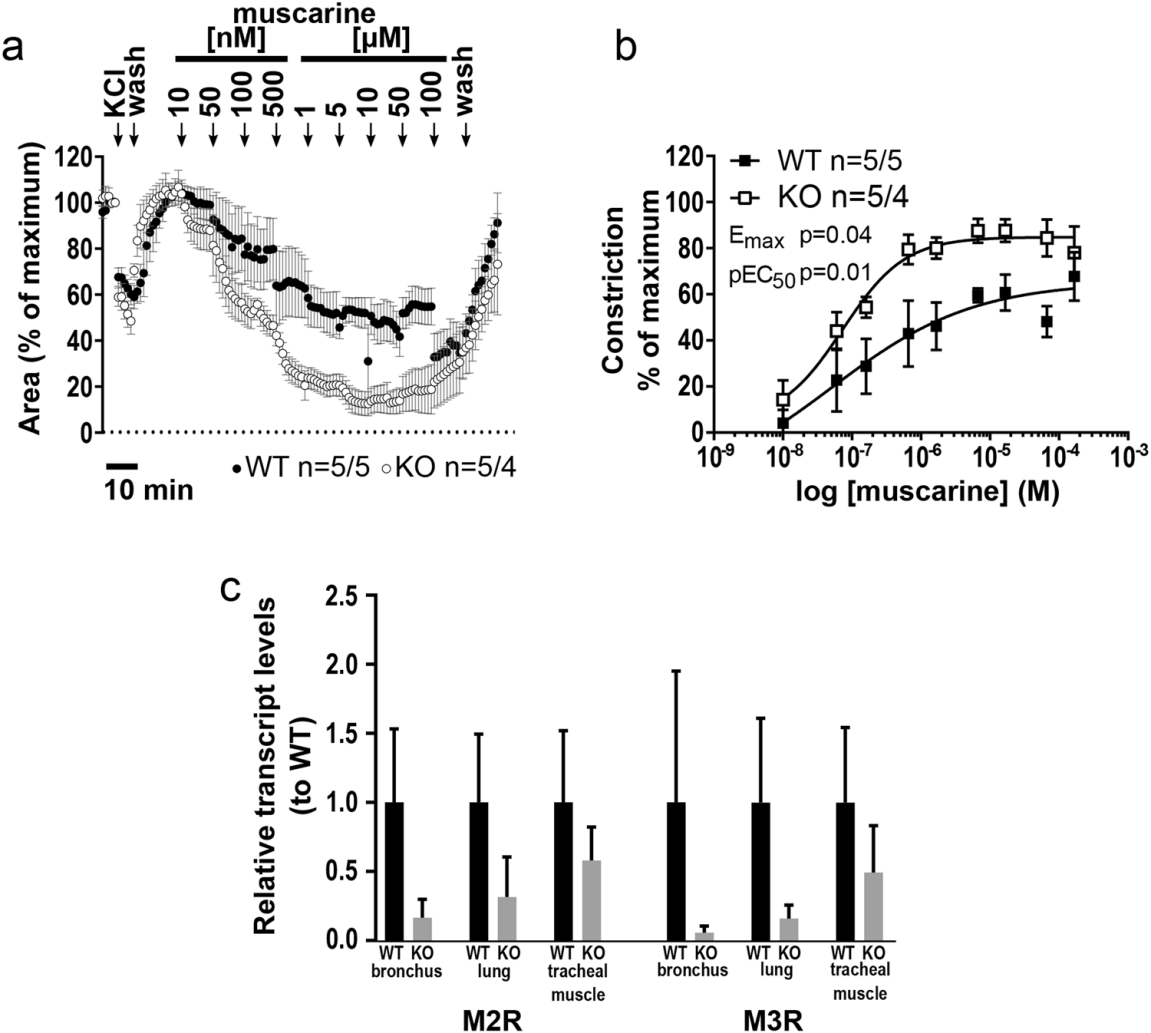
Bronchoconstrictor response to muscarine of peripheral bronchi from cav-3^+/+^ (WT) and cav-3^−/−^ (KO) mice. Videomorphometric analyses of PCLS, depicted are changes in the luminal area. Data are presented as mean of number of bronchi (n)/number of animals ± SEM. (**a**) Muscarine-mediated concentration-dependent decreases in the luminal airway area in cav-3^+/+^ and cav-3^−/−^ mice. KCl (60 mM) was applied as a viability control for 5 min, and the pre stimulus value was set as 100%. In response to different concentrations of muscarine (10 nM–100 µM) a dose dependent continuous contraction was observed in bronchi of cav-3^+/+^ and cav-3^−/−^ mice. (**b**) Depicted are the sigmoidal concentration-response curves (plotted using the Hill equation) of concentration versus luminal area reduction of peripheral bronchi of cav-3^+/+^ and cav-3^−/−^ mice. The Student’s unpaired t-test was used to analyze values for E_max_ and pEC_50_. Bronchi of cav-3^−/−^ mice responded with a stronger contraction (p ≤ 0.05) and a difference in pEC_50_ (p ≤ 0.05). (**c**) Relative transcript levels of muscarinic acetylcholine receptor subtypes 2 and 3 (M2R and M3R) genes standardized on internal β-microglobulin (β-MG) levels. Results for the cav-3^−/−^ tissues are presented relative to the results for cav-3^+/+^ tissues set to 1 to appreciate its potential differences independent from individual assay performance. Data are presented as mean ± SEM (Student’s t-test, *p < 0.05; n = 4 mice per genotype). No differences in M2R and M3R expression were observed between cav-3^−/−^ and cav-3^+/+^ tracheal SM, extrapulmonary bronchi and lung (with intrapulmonary bronchi).

Intrapulmonary bronchi exhibited a concentration-dependent muscarinic contraction measured as decrease in luminal airway area (Fig. 7a). At cumulative administration of muscarine, a significantly higher potency was observed in cav-3^−/−^ mice compared to cav-3^+/+^ mice (E_max_ = 88.93% vs. 65.08%, respectively, p = 0.036) (Fig. 7a,b). In addition to that pEC_50_ was significantly different between intrapulmonary bronchi from cav-3^+/+^ mice versus cav-3^−/−^ mice (p = 0.011, Fig. 7b). No significant changes in expression of M2R and M3R were detected in extrapulmonary bronchi, in lung tissue with intrapulmonary bronchi and in tracheal SM (Fig. 7c).

### 5-HT-induced contraction of intrapulmonary airway

**Figure 8:**
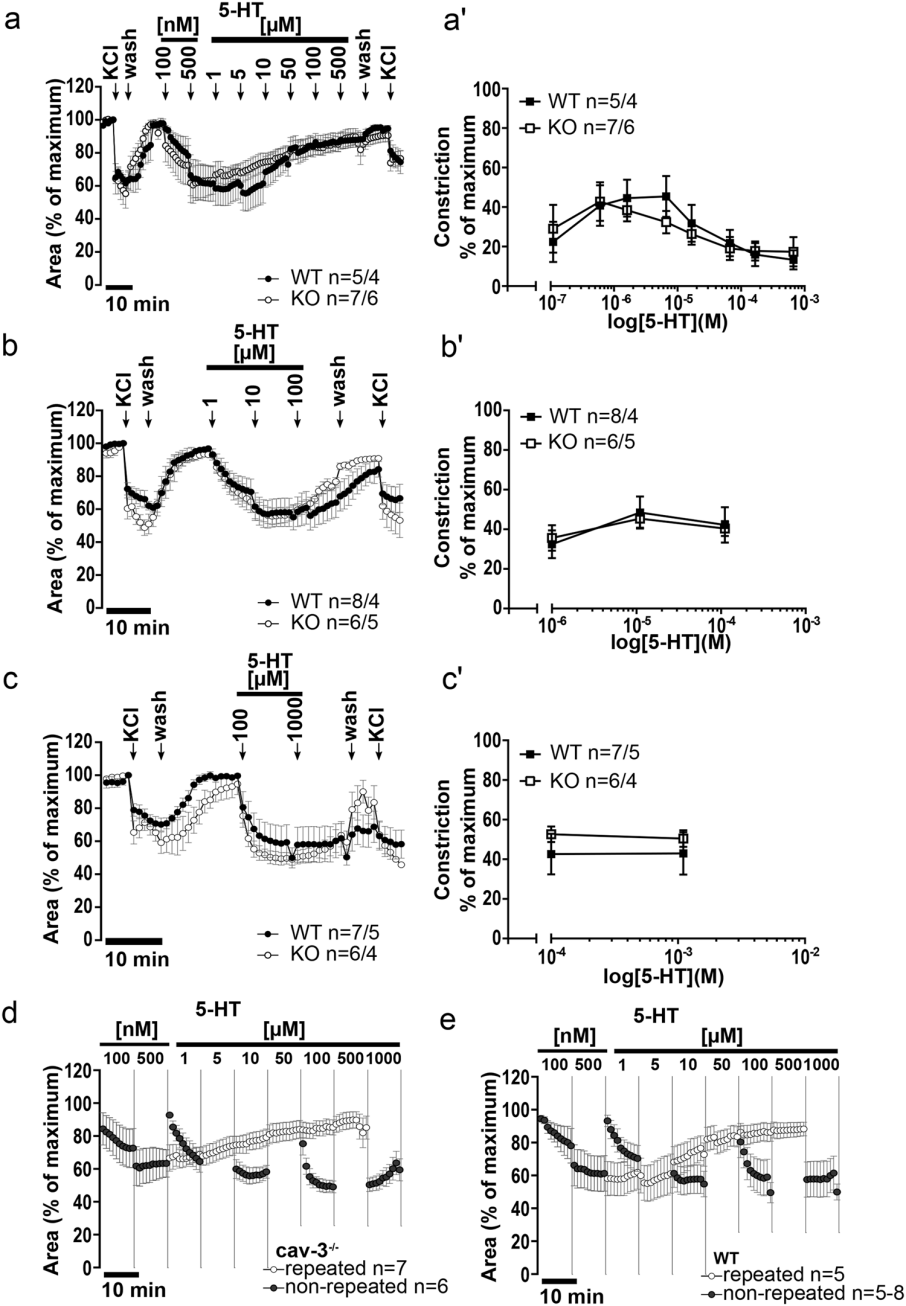
Bronchoconstrictor response to 5-HT of peripheral bronchi from cav-3^+/+^ (WT) and cav-3^−/−^ (KO) mice. Videomorphometric analyses of PCLS, depicted are changes in the luminal area. (**a**–**c**) Depicted are the 5-HT-mediated concentration-dependent changes in the luminal airway area in cav-3^+/+^ and cav-3^−/−^ mice. Data are presented as mean number of bronchi (n)/number of animals ± SEM. As a viability control, 60 mM of KCl was applied. The pre-stimulus value was set as 100%. (a’–c’) Concentration-dependent responses are shown as maximum reduction in the luminal area of mouse peripheral airways after administration of cumulative 5-HT concentrations. When data were analyzed with the Student’s unpaired t-test, at neither 5-HT application mode, a significant difference between the constrictor response from cav-3^+/+^ and cav-3^−/−^ mice was observed. (**d**,**e**) Comparison of the response to 5-HT after repetitive or non-repetitive stimulation in cav-3^−/−^ (**d**) and cav-3^+/+^ (**e**) mice. The intrapulmonary bronchi show a decrease of the bronchoconstriction mediated by 5-HT after repeated 5-HT application. All data are presented as mean number of bronchi (n)/number of animals ± SEM.

To test for a desensitizing effect of 5-HT during cumulative application, three different application schedules were chosen. Intrapulmonary bronchi exhibited a concentration-dependent contraction in response to stimulation with gradually increasing concentrations (100 nM–500 µM in half logarithmic scale) of 5-HT. Compared with bronchi from wild-type animals, cav-3 deficiency had no effect on the 5-HT-induced bronchoconstriction (Fig. 8a-a’). Intrapulmonary bronchi also exhibited a concentration-dependent contraction in response to less repetitive 5-HT application (1–100 µM) (Fig. 8b-b’). Compared with bronchi from wild-type animals, cav-3 deficiency had no effect on the 5-HT-induced bronchoconstriction. Stimulation with 100 µM 5-HT followed by application of the supramaximal concentration of 5-HT, 1 mM also resulted in a contraction (Fig. 8c-c’). Intrapulmonary bronchi responded to 100 µM 5-HT with an initial rapid constriction in both mouse strains and with a sustained constriction at 1 mM. Compared with bronchi from wild-type animals, cav-3 deficiency had no effect on the 5-HT-induced bronchoconstriction. Next, we compared repetitive 5-HT stimulation (100 nM–500 µM in half logarithmic scale) and the response to the same 5-HT-dose without cumulative stimulation (100 nM and 500 nM from cumulative doses of 100 nM–500 μM, 1 µM and 10 µM from 1–100 µM and 100 µM and 1 mM from 100 µM–1 mM). The contractor response was identical in cav-3^−/−^ as well in cav-3^+/+^ mice where single high doses of 5-HT led to a higher constriction compared to the same doses in cumulative stimulation. This left shift is indicative for desensitization of the response under repetitive stimulation with 5-HT (Figs 8d,e, S4).

### Immunofluorescence labelling for cav-3 in human lung

**Figure 9:**
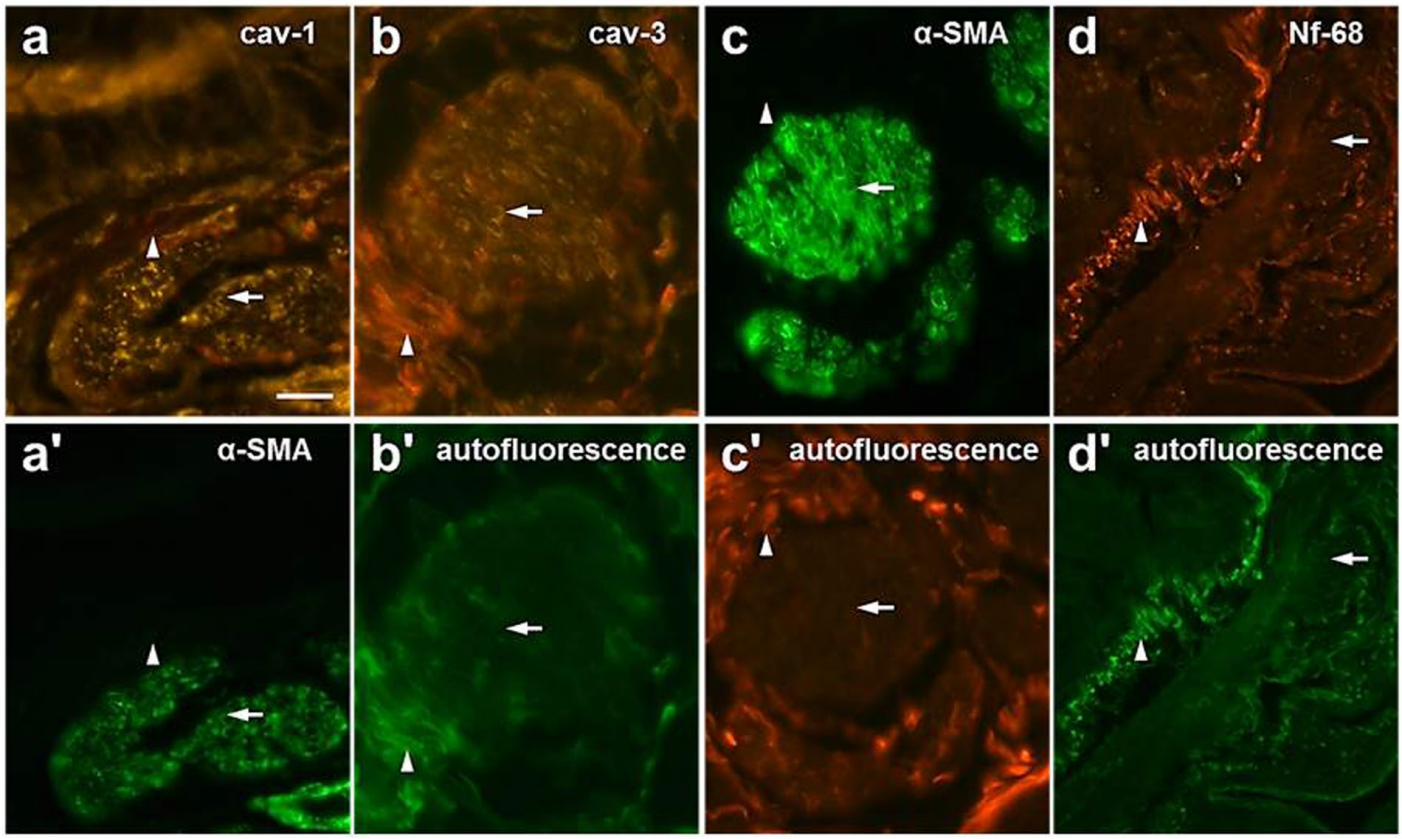
Immunofluorescence labelling for cav-3 in bronchial smooth muscle cells (SMC) of human lung. (**a**-a’) Cav-1 and α-SMA-immunoreactivity were colocalized in bronchial SMC (arrow). (**b**) Cav-3-immunoreactivity was also in found in bronchial SMC as evident by the α-SMA-labelling (arrow) in the same region in adjacent sections (**c**). (**d**) Isotype control was done by incubation with the monoclonal Nf-68-antibody. (a’-d’) The autofluorescence of the epithelial basal membrane and of connective tissue fibers is indicated by arrowheads. Bar = 75 µm.

In order to translate the findings from mice to humans, the protein expression of cav-3 was assessed by immunohistochemistry using monoclonal cav-3- and cav-1-antibodies. Human bronchial SMC were immunoreactive for α-SMA (Fig. 9c,a’). Immunofluorescence with antisera directed against α-SMA showed strong staining, while antibodies to cav-1 and cav-3 gave a faint staining (Fig. 9a,b) of the smooth muscle (Fig. 9a-a’,b-c’). The Nf-68 monoclonal antibody, isotype IgG2, was used as an irrelevant antibody isotype control for the monoclonal cav-3-antibody (Fig. 9d-d’).

## Discussion

Caveolae are defined by a set of core protein ingredients, cavs, which govern their structure and function^36^. In rat arterial SMC, a regulatory role of cav-1 and cav-3 in vascular contractility has been reported^37^. Confirming previous data from our group, cav-1 and cav-3 were identified in mouse and human ASM on protein level in the present study^13,32^. In contrast, an absence of cav-3 in isolated human ASM and in freshly dissected tracheal muscle was reported by other groups^20,38,39^. This discrepancy might be due to the different antibodies used for the detection and/or to alteration of cav-3 expression by the isolation procedure of the ASM. To address the functional role of cav-3 in ASM directly, we generated a caveolin-3-deficient (cav-3^−/−^) mouse strain. RT-PCR, Western blotting, and immunohistochemistry of a range of tissues confirmed that the cav-3 mRNA and protein are indeed absent in the cav-3^−/−^ mice. All cav-3^−/−^ mouse strains generated so far, including ours and those reported by Galbiati *et al*. and Hagiwara *et al*.^40,41^, are designed by targeting exon 2 of the cav-3 gene which encodes the bulk of the cav-3 protein with all of its functional domains. The most notable pathological findings in cav-3-deficiency were muscle fiber necrosis and mild myopathic changes^40–42^. Ultrastructural analysis of skeletal muscle from our cav-3^−/−^ mice also revealed mild myopathic changes with variability in the size of necrotic fibers in the cytoskeletal architecture of muscle tissue that is characteristic of muscular dystrophy (data not shown).

Cav-1/cav-3 were co-immunoprecipitated in extracts from lung and trachea in the present study. The protein expression of all other caveolins was maintained in all investigated airway and lung compartments in cav-3^−/−^ mice, suggesting that the other caveolar protein members are not dependent on cav-3 in ASM. Our quantitative data on the caveolar abundance marker, EHD-2, imply that cav-3 does not contribute to the abundancy of ASM caveolae, and caveolae are still present in cav-3^−/−^ mice. This is corroborated by electron microscopic analysis of tracheal SMC, revealing no alteration in caveolae numbers in cav-3^−/−^ mice. In contrast, cav-3 as shown in previous studies regulates caveolar formation in cardiac and skeletal muscle cells, indicating that the cav-3 deficiency leads to decreased numbers or the absence of caveolar structures with associated T-tubular disorganization and dramatic cardiomyopathy^40,41,43–45^. In addition, several studies using transgenic mice overexpressing cav-3 revealed an increased number of sarcolemmal muscle cell caveolae in skeletal muscle fibers and cardiomyocytes^46,47^. Thus, there are obvious differences between smooth and striated muscle, and it is expected that specific aspects of caveolar function rather than caveolar abundancy are dependent on the expression of cav-3 in tracheal SMC. Since we recently observed a decreased number of caveolae in tracheal SMC in cav-1^−/−^ mice in ultrastructural analysis, cav-1 appears to be crucial for caveolae abundancy and caveolar morphogenesis in tracheal SMC^12^.

ACh is the major physiological bronchoconstrictor and its effect is mediated primarily through the muscarinic ACh receptor subtype 3 (M3R) and, to a lesser extent, through the M2R subtype^35^. Several evidences accumulated that this muscarinic bronchoconstriction involves caveolae as signaling platforms^13^. Dysregulation of caveolae and caveolins is assumed to be involved in the pathogenesis of diseases associated with airway hyperreactivity^48^. In the past, a large number of signal transduction molecules were co-purified with cav-3, suggesting that cav-3 containing caveolae are involved in intracellular signaling^49^. Thus, knowing the regulatory role of cav-3 in the constrictor response to contractile stimuli may provide an opportunity to modulate bronchial hyperreactivity. The M2R directly interacts with cav-3, but not cav-1 in murine ASM, as shown by antibody-based confocal FRET analysis^13^. On this background, the prediction was that muscarinic bronchoconstriction would be diminished in a cav-3 gene deficient mouse. In contrast to this expectation, the response of all extrapulmonary airway segments (cranial to caudal trachea and extrapulmonary bronchi; assessed in organ bath recordings) to muscarine was indistinguishable in cav-3^−/−^ from cav-3^+/+^ mice. In intrapulmonary bronchi, assessed by videomorphometry of PCLS, however, our findings show a considerable increase rather than decrease in muscarinic bronchoconstriction in cav-3^−/−^ mice. This demonstrates an unexpected inhibitory regulatory role of cav-3 in cholinergic bronchoconstriction. In agreement with this finding, overactivity of SM was also reported in the rat urinary bladder with age and ascribed to a reduction in cav-3 protein expression^33,50^. The direct association between M2R and cav-3 in murine bronchial SMC^13^ suggests that this inhibition targets the M2R signaling pathway. The underlying molecular pathways are still unclear and cannot be finally deduced from the present data. Cav-3 may inhibit MR function by direct interaction, or organize MR-independent inhibitory signaling. Findings on other cellular systems point to a link to nitrergic signalling. Using FRET analysis, we have previously demonstrated a direct association of cav-3 and eNOS in rat airway epithelial cells^32^. In cardiac myocytes and in transfected COS-7 cells (fibroblast-like cell lines) coexpressing the M2R and eNOS, stimulation with carbachol leads to NO production^51–53^. In this setup, cav-3 suppresses basal eNOS activity but is indispensable for cholinergic activation of eNOS. The disruption of the inhibitory eNOS-cav-3 complex, e.g. with an oligopeptide corresponding to the cav-3 scaffolding domain, completely abrogated cholinergic stimulation of eNOS with subsequent elevation of cGMP levels^51,52^. In ASM, NO is a bronchodilator acting through cGMP-dependent and -independent mechanisms^54^, and NO production by ASM itself can be stimulated by the cholinergic agonist carbachol at low concentration acting via the M2R^55^. Hence, it seems to be a plausible scenario that cholinergic stimulation of ASM not only activates a strong constrictor pathway but also an inhibitory component involving M2R-cav-3-eNOS coupling. In such a setting, selectively removing cav-3, as it is the case in cav-3 deficient mice, would abrogate the inhibitory component, resulting in enhanced cholinergic constriction, whereas disruption of the entire signaling platform by MCD treatment would also affect the constrictor component, as reported earlier^13^. This would imply that cav-3 is either not involved at all in this constrictor response or at least dispensable and its function might be compensated by cav-1 in this pathway. Indeed, we also observed a hetero-oligomeric complex of cav-1 and cav-3 in trachea and lung by Co-IP. Several proteins binding to cav-3 are also able to interact with cav-1^25,56^, suggesting that cav-3 might be exchangeable with cav-1. Considering the dependency of muscarinic constriction on intact caveolae, further functional studies with cav-1/cav-3-deficient mice or cavin-1-deficient mice which exhibit phenotypes reminiscent of cav-1 and cav-3-deficient mice^57^ are necessary to solve this.

Notably, this augmentation of cholinergic bronchoconstriction in cav-3 deficiency was not noted in organ bath recordings from extrapulmonary airways. This difference to intrapulmonary airways in PCLS may be due to different receptor coupling in extra- versus intrapulmonary airways. Of course, it also cannot be excluded that the different methodological approaches to record ASM constriction - force recording in organ bath versus videomorphometry of PCLS - may have contributed substantially to this difference.

The effects of 5-HT on ASM tone are complex. Both relaxant and constrictor activities were described depending on the concentrations and the species that had been used. In mice, different modes of action of 5-HT have been reported for extra- and intrapulmonary airways, respectively. In the trachea, 5-HT is assumed to cause release of ACh either from epithelial cells^58^ or from cholinergic nerve fibers^59,60^ which then, in turn, causes airway constriction. In intrapulmonary airways, however, serotonergic bronchoconstriction is independent from the presence of M3R and, therefore, not indirectly mediated via ACh^7^. Similarly, marked differences between extra- and intrapulmonary airways were also noted in the present study, in that the 5-HT-mediated constriction was equally strong in intrapulmonary airways of knock-out and wild-type mice while it was absent in extrapulmonary airways of cav-3^−/−^ mice. Serotonergic airway constriction in murine extrapulmonary airways is considered to be indirectly mediated through ACh release from epithelial cells or nerve fibers^58–60^. Since muscarinic constriction of extrapulmonary airways was unaffected in cav-3^−/−^ mice, the cholinergic component of this indirect effect should not be responsible for the abrogated serotonergic effect. Among the suspected immediate targets of 5-HT, cholinergic neurons and epithelial cells, there is no clear evidence for cav-3 expression in autonomic neurons, although our group has previously reported cav-3 expression in sensory neurons^61^. However, we here directly observed cav-3 in tracheal epithelial cells, consistent with earlier reports of our group in rat^62^. Notably, we did not observe cav-3-immunoreactivity in the epithelium of intrapulmonary bronchi, which correlates with the unaffected serotonergic response at this airway level.

In intrapulmonary bronchi, a large portion of the reaction to 5-HT is due to direct action upon the smooth muscle. In a parallel study we showed expression of the 5-HT receptor subtypes 5-HT1B, 5-HT2A, 5-HT6 and 5-HT7 in murine lung, and the 5-HT2A receptor inhibitor ketanserine inhibited 5-HT-induced constriction in murine PCLS^12^. Also, in other species the 5-HT2A receptor has been linked to direct serotonergic smooth muscle constriction^9,63^. The 5-HT2A receptor was shown to be located in caveolar and non-caveolar fractions in bovine ASM and in cardiomyocytes. In cardiomyocytes, cav-3 regulates its shift between caveolar and lipid raft membrane compartments^28,30,49^. There, it associates with the 5-HT2A receptor and negatively regulates the hypertrophic response of cardiomyoblasts and neonatal cardiomyocytes to 5-HT^30^. Despite this known interaction of cav-3 with 5-HT2A receptors, cav-3 deficiency did not affect serotonergic intrapulmonary bronchoconstriction in this study, whereas disruption of the entire signalling platform by MCD treatment entirely abrogated it, as reported earlier^13^. This implies that a cholesterol-rich membrane domain, most likely a caveolae is needed for serotonergic bronchoconstriction, but cav-3 is not involved at all in this response or at least dispensable. Finally, our results indicate a stimulating role for cav-3 in serotonergic bronchoconstriction of extrapulmonary airways and an inhibiting role for cholinergic constriction of intrapulmonary bronchi. As such, the recognition of a role of cav-3 in modulating cholinergic and serotonergic responses may provide new therapeutic targets in the treatment of airway hyperreactivity.

## Materials and Methods

### Mouse genetics and husbandry

To generate cav-3 knock-out mice, embryonic stem cells (ESC) were transfected with targeting vector and screened for homologous recombination by the PolyGene Company (Zurich, Switzerland). They produced the floxed germline within the knock-out mouse project 8053. Briefly, the targeting vector expressing neomycin resistance gene (neo^R^) flanked by two FRT sites was placed in an upstream of exon-2 which itself was flanked by two LoxP sites (Supplement Fig. S1). ESC were electroporated and the transfected clones were screened to identify the targeted vector. Resistant colonies that contained neo^R^ were tested for homologous recombination and the resistant colonies of transfected ESC were used to produce a chimeric mouse (8052.1007).

We used these cav-3-loxP (floxed) male mice which shows FLP mediated deletion of the FRT-flanked neo^R^ cassette to establish a conditional cav-3 knock-out mouse colony. By crossing floxed chimeric animals to the C57BL/6 J Cre deleter mouse strain from Jackson to obtain cav-3 knock-out and Cre negative homozygous mice. The loss of the genomic locus of interest was confirmed by genotyping (Supplement Fig. S2), immunohistochemistry and Western blot. Genotyping was carried out by a multiplex PCR with primers shown in Supplemental Table S1. Immunohistochemistry, real-time PCR, western blotting, Co-IP, electron microscopy and functional experiments were performed on 12–22-week-old cav-3^−/−^ (n = 42) mice and on the corresponding cav-3^+/+^ mice (n = 40) kept under specified pathogen-free (SPF) condition. The cav-3-deficient mice and the corresponding wild-type mice used in all experiments were sex and age matched. The animals were held according to the German guidelines for the care and use of laboratory animals. Animal experiments were approved by the local committee at the Regierungspräsidium Giessen, Hesse, Germany (permit Nr. JLU-Nr. 491) and by the Saarland’s institutional Animal Care and Use Committee. For videomorphometric analysis, mice were killed by cervical dislocation. Otherwise, all animals were killed by inhalation of an overdose of isoflurane and exsanguination (Abbott, Wiesbaden, Germany).

### Substances and Antibodies

The following substances were used: muscarine and serotonin hydrochloride (5-Hydroxytryptamine hydrochloride, 5-HT, H9523) were purchased from Sigma-Aldrich (Munich, Germany). Muscarine and 5-HT were dissolved in water at 10 mM and diluted in HEPES-Ringer buffer to the desired experimental concentration immediately before use. Antibodies and their sources were as follows: FITC-conjugated anti-α-smooth muscle actin (anti-α-SMA; IHC human 1:1000), monoclonal from mouse (clone 1A4; Sigma, Taufkirchen, Germany); anti-cav-1 amino-terminal (N-20) (IHC human 1:400, IHC mouse 1:500, Western blotting 1:400, Co-IP 1 µg/µl), polyclonal from rabbit (sc-894; Santa Cruz Biotechnology, USA); anti-cav-3 (IHC human 1:500), monoclonal from mouse (clone 610421/26, isotype IgG2; BD Bioscience, Germany); goat anti-cav-3 amino-terminal (N-18) (IHC mouse 1:300, Western blotting 1:500), polyclonal from goat (sc-7665; Santa Cruz Biotechnology); anti-EHD2 (Western blotting 1:500), polyclonal from goat (Ab23935; Abcam, UK); anti-SDH-A (Western blotting 1:500), polyclonal from rabbit (code 11a; own laboratory); anti-β-tubulin IV (Western blotting 1:400) monoclonal from mouse (clone Mu178-UC/ONS1A6; Biogenex, USA); anti-cav-1 + cav-3 (IHC human 1:400), polyclonal from rabbit (63941; BD Bioscience, Germany); anti-neurofilament-68 (Nf-68) (IHC human 1:300), monoclonal from mouse (clone N5139/Nr4, isotype IgG2; Sigma). Secondary antibodies used for IHC in this study were Cy3-conjugated donkey anti-rabbit-Ig (1:1000; AP182C Chemicon, Germany), Cy3-conjugated donkey anti-goat-Ig (1:800; 705-165-003 Dianova, Germany), and Cy3-conjugated donkey anti-mouse-Ig (1:1000; 715-165-150 Dianova). Secondary antibodies used in this study for Western blotting horseradish peroxidase-conjugated goat anti-rabbit-Ig (1:10000; 31460 Thermo Scientific, USA); goat anti-mouse-Ig (1:10000; M32307 Thermo Fisher, Germany) and rabbit anti-goat-Ig (1:5000; 61–1620 Thermo Fisher).

### Western blotting

The tissues were removed and immediately frozen on dry ice and either processed the same day or stored at −80 °C. In addition bronchus and tracheal different parts including tracheal muscle, abraded tracheal epithelium and the rest of the trachea (epithelium and muscle were removed) from 6 pooled cav-3^+/+^ or cav-3^−/−^ mice were shock-frozen and stored at −80 °C until use. Samples were homogenized with a homogenizer (Retsch MM300, California, USA) in octylglucoside lysis buffer (10 mM Tris-HCl, pH 7.4, 50 mM NaCl, 60 mM octylglucoside, and 1% Triton X100) (Sigma-Aldrich) and supplemented with a concentrated complete mini protease inhibitor cocktail (Roche Diagnostics, Mannheim, Germany) for 10 min.

The homogenate was cleared by centrifugation at 8000 rpm for 5 min. The protein concentration in each tissue was determined using the method by Lowry with a commercially available kit (Bio-Rad, Hercules, CA, USA). Then 50 μg of each protein lysate supernatant were mixed with reducing sample buffer, boiled and run on SDS-PAGE separating gel. For the caveolin protein with a low molecular weight, a 15% SDS-PAGE separating gel, and for EHD2, β-tubulin IV and SDH-A a 10% SDS-PAGE separating gel was used. For the 15% SDS-PAGE separating gel, 7.5 ml of 30% acrylamide (Roth, Karlsruhe, Germany), 2.8 ml of 2 M Tris-HCl, pH 8.8; 75 µl of 20% SDS (Serva, Heidelberg, Germany), 80 µl 10% ammonium persulfate (Merck, Darmstadt, Germany), 7.5 µl TEMED (Roth, Karlsruhe, Germany) and 4.62 ml H_2_O were poured. For the 10% SDS-PAGE separating gel, 5 ml of 30% acrylamide, 2.8 ml of 2 M Tris-HCl (pH 8.8), 75 µl of 20% SDS, 80 µl 10% APS, 7.5 µl TEMED and 7.12 ml H_2_O were mixed. After the complete polymerization of the separating gels, 1 ml 30% acrylamide, 50 µl of 20% SDS, 1.25 ml of 1 M TRIS-HCl (pH 6.8), 80 µl 10% APS, 10 µl TEMED and 7.7 ml H_2_O were poured to make the stacking gel. The gels were blotted using a semi-dry blotter (Peqlab GmbH, Erlangen, Germany) and the polyvinylidine difluoride membranes (Millipore, Schwalbach, Germany) were blocked in a TTBS solution (0.01 M Tris-HCl (pH 8.0), 0.2 M NaCl and 0.05% Tween-20) containing 10% dried skimmed milk powder for 3 h and incubated overnight with the appropriate antibodies washed and incubated with secondary horseradish peroxidase-conjugated antibodies. Immunoreactive bands were visualized by the enhanced chemiluminescence kit (Thermo Scientific, USA) with Fusion FX (Vilber Lourmat, Australia). To determine the fold differences in protein expression the densitometry data were used. The intensities of protein bands were quantified using image J analysis program (Bethesda, Maryland, USA). Protein expression level was quantified with reference to tubulin control.

### Electron microscopy

All electron microscopy experiments were performed as described previously^12^. Briefly, tracheae of cav-3^−/−^ and cav-3^+/+^ mice were dissected, embedded, fixed and subsequently cut with a final thickness of approximately 80 nm on an ultramicrotome (Reichert Ultracut E, Leica, Bensheim, Germany), stained with alkaline lead citrate, and examined with an EM 902 transmission electron microscope (Zeiss, Jena, Germany).

### Real-time RT-PCR

Tracheal muscle, whole trachea, extrapulmonary bronchi, intrapulmonary bronchi/lung and pieces of peripheral lung were obtained from cav-3^+/+^ (cav-3-floxed mice) and cav-3^−/−^ mice (>3 month old, n = 8). The tracheal SM was dissected as a strip from the dorsal wall of the trachea after the removal of the epithelium. The lack of epithelium was confirmed under microscopical observation. The samples were either shock-frozen in RLT buffer plus (Qiagen, Hilden, Germany) and stored at −80 °C until use or immediately processed for RNA isolation. RNA was isolated and reverse transcribed as described previously^12^. Real-time PCR was performed in an iCycler (Bio-Rad, Munich, Germany, cf. data sets presented in Figs 2d and 3d) using iTaq Universal SYBR Green Supermix (Bio-Rad Laboratories Inc., Hercules, CA, USA). The PCR conditions included initial denaturation for 10 min at 95 °C followed by 40 cycles of 30 s at 95 °C, 30 s at 60 °C, and 30 s at 72 °C. Quantitative PCR (qPCR) had been performed using oligo pairs that allow to test for the transcript levels of the target genes cav-1, EHD2, M2R, M3R, 5-HT1AR, 5H1B, 5H2AR, 5-HT6R, 5-HT7R, and the reference genes β-actin and β-2-microglobulin (β-MG). All gene-specific primer sets are given in Table 1. M2R, M3R, cav-1, EHD2, β-actin and β-MG and oligos have been purchased from MWG Biotech (Ebersberg, Germany) all other oligos were purchased from IDT (Coralville, Iowa, USA). All qPCR reactions were run as duplicates on a CFX Connect System (Bio-Rad Laboratories Inc., cf. data sets presented in Figs 6e and 7c) with adjusted settings for the recommended SYBR Green Supermix. For M2R, M3R, 5H1B, 5H2AR and β-MG we need to note that in most cases all four samples per group (cav-3^−/−^ vs. cav-3^+/+^) yielded a call in qPCR performance. In a few cases at least three samples have been used to calculate our results. The amplification curve was collected, and the relative transcript level of the target mRNA in each sample was calculated by normalization of Ct values to the reference mRNA (β-MG) using the following equation: V = 2^CT[reference]^/2^CT[target]^. V is the relative value of target gene normalized to the reference. For comparison between cav-3^−/−^ vs. cav-3^+/+^, target expression in cav-3^−/−^ was normalized to cav-3^+/+^ set to 1. Statistical analyses were performed using Student’s *t*-test; data are presented as mean ± SEM (***p < 0.001; n = 4 mice per genotype).

**Table 1:** Oligonucleotide primers for PCR analysis.

Gene	Genebank accession No.	Primer	Product length (bp)
β-actin	NM007393.3	fwd gtgggaatgggtcagaagg rev ggcatacagggacagcaca	299
β-2-Microglobulin (β-MG)	NM009735	fwd attcacccccactgagactg rev gctatttctttctgcgtgcat	192
cav-1	NM001243064.1	fwd gcacaccaaggagattgacc rev agatgagtgccattgggatg	212
EHD2	NM153068.3	fwd tggagagcatcagcatcatc rev gtgggcatcaaagagcaaga	140
5-HT1AR	NM008308.4	fwd tctctccctccctcttgctc rev aattccagggcaccataacc	133
5-HT1BR	NM010482	fwd aagccaaagcagaggaggag rev cggtcttgttgggtgtctgt	177
5-HT2AR	NM172812.1	fwd atagccgcttcaactccaga rev tcatcctgtagcccgaagac	106
5-HT6R	NM021358.2	fwd ggtgccatctgcttcaccta rev gcagccaggtgacaaagaac	250
5-HT7R	NM008315	fwd gccacttcttctgcaacgtc rev ttcacattctgagcccatcc	226
M2R	NM203491.3	fwd gaatggtgatgaaaagcaga rev gcagggtgcacagaaggtat	193
M3R	NM033269.4	fwd cacagccaagacctctgaca rev atgatgttgtagggggtcca	222

For cav-1 and EHD2 analyses were done in triplicate and the mean cycle thresholds (CT) for all target genes were calculated. The ΔCT of all target genes compared with β-actin was calculated as follows:

$${\rm{\Delta }}\text{CT}\,{\rm{target}}\,{\rm{gene}}={\rm{CT}}\,{\rm{target}}\,{\rm{gene}}-{\rm{CT}}\,{\rm{\beta }} \mbox{-} \text{actin}.$$

Control reactions included always the absence of DNA template, the absence of reverse transcriptase enzyme and brain as a positive control tissue.

### Immunohistochemistry

The thorax of the mouse was opened and a plastic cannula (1.1 mm diameter; Braun, Melsungen, Germany) was inserted into the trachea. After placing the cannula and fixing it, the lungs were filled with approximately 2 ml of OCT compound (Sakura, Zoeterwoude, Netherlands) diluted with an equal amount of 0.1 M phosphate buffer (pH 7.4). Then, all thoracic viscera (heart, lungs and trachea) were removed, frozen on filter paper in melting isopentane, transferred to liquid nitrogen, and finally stored at −80 °C.

Cryosections (10 μm) were cut, fixed with acetone at −20 °C for 10 min, air-dried, and incubated for 1 h in 50% horse serum in 0.005 M phosphate-buffered saline (PBS). Primary antibodies were diluted in PBS with addition of 0.01% NaN_3_ and 0.05 M NaCl and applied overnight at room temperature. After a washing step with PBS, the sections were incubated with Cy3-coupled secondary antibody for 1 h at room temperature. The slides were rinsed with PBS, postfixed for 10 min in 4% PFA solved in PBS, rinsed again, and coverslipped with carbonate-buffered glycerol (pH 8.6). The sections were inspected with an epifluorescence microscope (Axioplan, Zeiss) using appropriate filter sets. Lung biopsies from donors (n = 2) were provided by the Institute of Pathology and Cytology, Wetzlar (Prof. Ludger Fink). Bronchial samples were fixed in 10% formalin according to the standard method and they were embedded in paraffin. The study was approved by the responsible ethic committee (Ref. No. 100/07).

The paraffin embedded human lung tissues were cut at a thickness of 8 µm and sections were dried overnight in an incubator (Heraeus, Hanau, Germany) at 37 °C. Sections were stored at room temperature until use. Before incubation with the primary antibody, the sections were deparaffinised (in xylene, absolute, 96%, 80%, 70% and 50% ethanol, respectively, for 5 min). Next, microwave oven heating for antigen retrieval in PBS containing 10% citric acid, pH 6 (Merck, Darmstadt, Germany) was done and the slides were incubated for 1 h in 10% normal horse serum, 0.5% Tween-20, 0.1% BSA in PBS, pH 7.4 before applying the appropriate primary antibody. The appropriate primary antibodies were applied either singly or in combination with anti-α-smooth muscle actin for double-labelling immunofluorescence overnight at room temperature (RT). Secondary antibody were incubated with for 1 h at RT. Finally, the slides were postfixed for 10 min in 4% PFA and coverslipped with carbonate-buffered glycerol (pH 8.6). A monoclonal antibody, isotype IgG2, raised against neurofilament-68 (Nf-68) was used as an irrelevant antibody isotype control for the monoclonal cav-3 antibody on sections from paraffin embedded specimens.

### Co-Immunoprecipitation

Different tissue samples from both cav-3^+/+^ and cav-3^−/−^ mice were homogenized with a homogenizer in octylglucoside lysis buffer supplemented with concentrated protease inhibitor cocktail. Skeletal muscle or heart tissue from cav-3^+/+^ mice were used as positive control. The homogenate was cleared by centrifugation as described for Western blotting. Total tissue homogenate (2 mg) for each reaction was incubated with 1 µg of anti-cav-1 antibody in 150 µl PBS + Tween-20 (0.02%) at 4 °C and rotated overnight. Then, we added 50 µl dynabeads coated with protein G (Life Technologies GmbH, Darmstadt, Germany) to each immunoprecipitation (IP) tube and rotated them for 4 h at 4 °C. Next, we removed the supernatant and froze the samples for further analysis. The dynabeads-antibody-protein complex was washed three times with 200 µl PBS + Tween-20. Then, PBS + Tween-20 was added to the dynabeads-antibody-protein complex and it was transferred to a fresh tube.

We put the fresh tube with dynabeads-antibody-protein complex on a magnetic base and removed the PBS. Then, we added reducing sample buffer and boiled at 95 °C for 5 min, followed by gel electrophoresis on a 15% separating acrylamide gel. Western blot analysis was carried out as explained above.

### Videomorphometry

To study the bronchoconstrictor response of intrapulmonary airways with preserved morphology we performed videomorphometric recordings^12^. PCLS were prepared as described previously^13,64^. PCLS were incubated in minimal essential medium (MEM, GIBCO, Karlsruhe, Germany) under continuous normoxic gas supply, so that the lung sections were constantly floating in the medium. After 2 hours, the “agarose plaques” filling the airspaces of the bronchi that were studied (with luminal area of 150–250 µm) had vanished. Nonetheless, we cannot exclude that some agarose is still present in smaller airways and in the alveoli (for preparing PCLS and avoiding any agarose left in the lung see^65^). Bronchoconstriction and dilatation were given as a percentage of the initial bronchial luminal area. Only bronchi responding to the control stimulus KCl with 20% constriction were considered for further analysis. To address the possibility that the prolonged or repeated application of 5-HT might lead to sensitization or desensitization of 5-HT receptors, we used different 5-HT application modes. The following experimental designs for muscarine and 5-HT administration were applied:

(A) KCl (5 min), wash (15 min), additive ascending concentrations of muscarine (10 nM, 50 nM, 100 nM, 500 nM, 1 μM, 5 μM, 10 μM, 50 μM, 100 μM; 10 min per concentration), wash (10 min).
(B) KCl (5 min), wash (15 min), additive ascending concentrations of 5-HT (100 nM, 500 nM, 1 μM, 5 μM, 10 μM, 50 μM, 100 μM, 500 μM; 10 min per concentration), wash (10 min), KCl (viability control; 5 min).
(C) KCl (5 min), wash (15 min), additive ascending concentrations of 5-HT (10 nM, 100 nM, 1 μM, 10 μM, 100 μM, 1 mM; 10 min per concentration), wash (10 min), KCl (5 min).
(D) KCl (5 min), wash (15 min), additive ascending concentrations of 5-HT (1 μM, 10 μM, 100 μM; 10 min per concentration), wash (10 min), KCl (5 min).
(E) KCl (5 min), wash (15 min), additive ascending concentrations of 5-HT (100 μM, 1 mM; 10 min per concentration), wash (10 min), KCl (5 min).

### Organ bath force recordings

Organ bath force recordings were performed as described previously^12^. Briefly, the surrounding tissue was removed from the trachea and the trachea was divided into 3 pieces, spanning 4 cartilage rings each. The left extrapulmonary bronchus was dissected separately. Then, the pieces have been fixed with two clips in the organ baths system (ADInstruments, Heidelberg, Germany) that was connected to a computer and isometric contraction was recorded. The medium in the chambers consisted of MEM (Invitrogen Gibco, Oslo, Norway) supplemented with 1% penicillin/streptomycin (PAA Laboratories, Coelbe, Germany), gassed with a mix of 95% O_2_/5% CO_2_ and heated to 37 °C.The upper clip was connected to an isometric force transducer (Power Lab 8.30; ADInstruments GmbH). First, 1 g of a passive load was used to equilibrate all tissue rings and then 0.5 g tension was used to adjust the tissue. The transducer converted changes in the isometric contraction into an amplified DC output voltage that was recorded with the LabChart 6 (ADInstruments) software.

After an equilibration period (ca. 30 min) until the samples established a stable baseline tension, KCl (60 mM) was applied for estimation of the reference response. After a washing step with fresh MEM medium, cumulative ascending doses of 5-HT or muscarine were applied. Tension changes have been measured as force [in grams] and recorded using software. The experiments were designed in the following way:

1. KCl (5 min), wash (15 min), additive ascending concentrations of muscarine (1 nM, 5 nM, 10 nM, 50 nM, 100 nM, 500 nM, 1 μM, 5 μM, 10 μM, 50 μM; 10 min per concentration), and wash (20 min) or
2. KCl (5 min), wash (15 min), additive increasing concentrations of 5-HT (10 nM, 50 nM, 100 nM, 500 nM, 1 μM, 5 μM, 10 μM, 50 μM, 100 μM, 500 μM, 10 min per concentration), wash (15 min) and KCl.

Each experiment was repeated with tissues from at least four animals. Respective numbers of animals are depicted in each graph.

KCl was used for estimation of the receptor-independent contraction. The reactivity of the response to muscarine or 5-HT was calculated as follows:

$${\rm{Reactivity}}\,[{\rm{muscarine}}]=\,{\rm{Max}}\,{\rm{force}}\,[{\rm{muscarine}}]/{\rm{Max}}\,{\rm{force}}\,[{\rm{KCl}}]$$

or

$${\rm{Reactivity}}\,[5 \mbox{-} \text{HT}]=\,{\rm{Max}}\,{\rm{force}}\,[5 \mbox{-} \text{HT}]/{\rm{Max}}\,{\rm{force}}\,[{\rm{KCl}}].$$

### Statistical analysis

The data regarding the time courses and dose responses are shown in the graphs as mean ± standard error of the mean (SEM). The GraphPad Prism software version 7 (La Jolla CA, USA) was used for statistical analyses. Normal distribution was analyzed in all data sets with the Kolmogorov-Smirnov or the Shapiro-Wilk tests. According to the results of normally distributed data were further analyzed with parametric tests: Student’s unpaired t-test or One-Way ANOVA followed by Dunnett’s multiple comparisons test. Data that did not show normal distribution were subjected to nonparametric tests: Mann-Whitney U-test or Kruskal-Wallis test followed by Dunn’s multiple comparisons test. The maximal effects (E_max_) and the pEC_50_ values of the responses to the agonists were calculated with nonlinear regression sigmoidal curve analysis according to the Hill equation. Differences between values were considered to be statistically significant when *P* ≤ 0.05.

## Electronic supplementary material

Supplementary information
