# Supplementary material for: Caveolin-3 differentially orchestrates cholinergic and serotonergic constriction of murine airways
Source: Sci Rep. 2018 May 14;8:7508. doi: 10.1038/s41598-018-25445-1 (PMC5951923; doi:10.1038/s41598-018-25445-1)
Supplement: Supplementary file 1 — Supplementary information [file 41598_2018_25445_MOESM1_ESM.pdf]

Caveolin-3 differentially orchestrates cholinergic and serotonergic constriction of murine airways

Keshavarz M[1], Skill M[1], Hollenhorst MI[3], Maxeiner S[3], Walecki M[1], Pfeil U[1], Kummer W[1,2], Krasteva-Christ G [1,2,3#, *]

[1]Institute of Anatomy and Cell Biology, Justus-Liebig-University Giessen, Germany,

[2]German Center for Lung Research (DZL), Germany,

[3]Institute of Anatomy and Cell Biology, School of Medicine, Saarland University, Germany.

# present address

* Correspondence to: gabriela.krasteva-christ@uks.eu

Supplementary information

Genotyping DNA extracted from tail snips was analyzed by PCR with different primer sets detecting the wild-type and knock-out version of the cav-3 gene. Samples from animals with the wild-type, heterozygous and loxP-flanked cav-3 gene showed a band of the predicted size, and samples from animals that carried the knock-out gene did not present any band (Fig. S2A). To confirm the presence of the knock-out allele, we did another PCR with a different primer set, and all genotypes of the cav-3 gene demonstrated a band of the predicted size (Fig. S2B). Homozygous cav-3 knock-out and wild-type mice were also tested for Cre transgene and those with deleted Cre were selected for mating and all sets of experiments (Fig. S2C). Negative controls were run by adding H2O instead of the DNA template.

Fig. S1 Schematic overview of the generation of cav-3-loxP mouse strain and of general cav-3 deficient mice. Neo^R flanked by two FRT sites was placed upstream of exon-2 which itself was flanked by two loxP sites. Mating of FLIP deleter mice with cav-3-loxP/neoFRT mice caused depletion of the neo^R locus and generation of cav-3-loxP mice. Further mating of cav-3-loxP (Flox) mice with Cre deleter mice caused depletion of the cav-3 exon-2 locus and generation of cav-3 deficient mice.

Fig. S2 PCR genotyping of cav-3 knock-out mice. The cav-3 knock-out mice were generated as described in the materials and methods section. The sequences of the primers are shown in Supplementary Tab. S1. DNA extracted from tail cuts was analyzed by PCR using different primer sets. (A) 5/6 primers detected wild-type (w/w) and loxP-flanked cav-3 gene (p/p) with amplicons of 323 and 368 base pairs and no amplicon for cav-$3^{-/-}$ (d/d) mice. (B) Analyses with 5/12 primers detected w/w, p/p and d/d alleles of the cav-3 gene with 1700, 2000 and 497 bp, respectively. Heterozygous p/w showed bands for wild-type and loxP-flanked alleles using both 5/6 and 5/12 primers. (C) PCR analysis with Cre primers showed animals carrying the Cre allele (+). Control reaction included the absence of template (H_2O). M = marker.

Fig. S3 Cav-3 interact with cav-1 in cav-$3^{+/+}$ (WT) mice. Immunoprecipitates (IP) with cav-1 are immunoblotted (IB) for cav-3 using a series of increasing exposure times. Positive CO-IP controls include heart and skeletal muscle from cav-$3^{+/+}$ mice. Lung and trachea input as intact protein lysate from cav-$3^{+/+}$ mice is blotted and the expression level of cav-3 protein is observed with longer exposure time. The negative control of CO-IP includes beads and antibody in the absence of lysate (no sample).

Fig. S4 Comparison of the response to 5-HT after repetitive stimulation with the response to 5-HT after non-repetitive stimulation of cav-$3^{-/-}$ (A) and cav-$3^{+/+}$ (B) mice. The intrapulmonary bronchi show a decrease in 5-HT-induced bronchoconstriction after repetitive 5-HT application. Data are presented as mean of number of bronchi (n) / number of animals ± SEM.

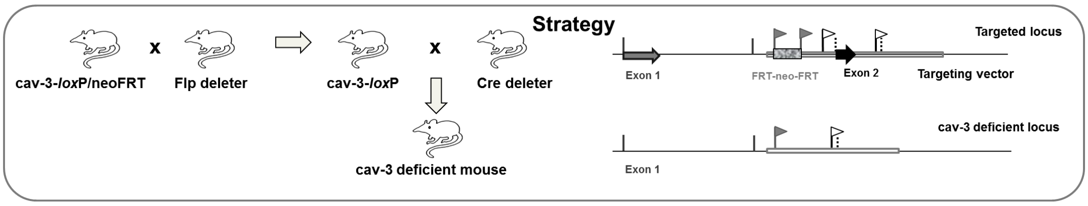

Fig. S1 Schematic overview of the generation of cav-3-loxP mouse strain and of general cav-3 deficient mice.

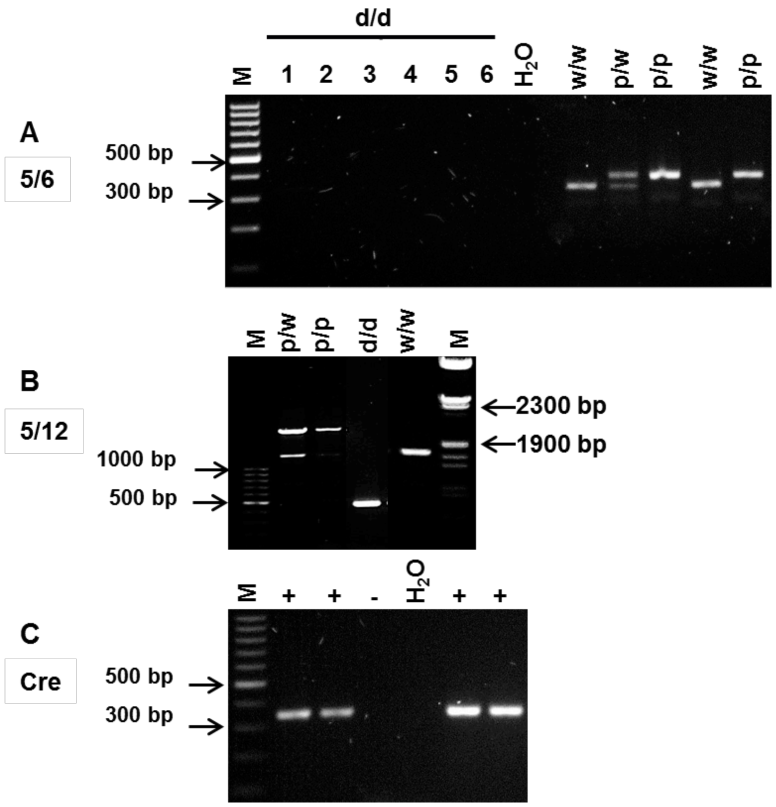

Fig. S2 PCR genotyping of cav-3 knock-out mice.

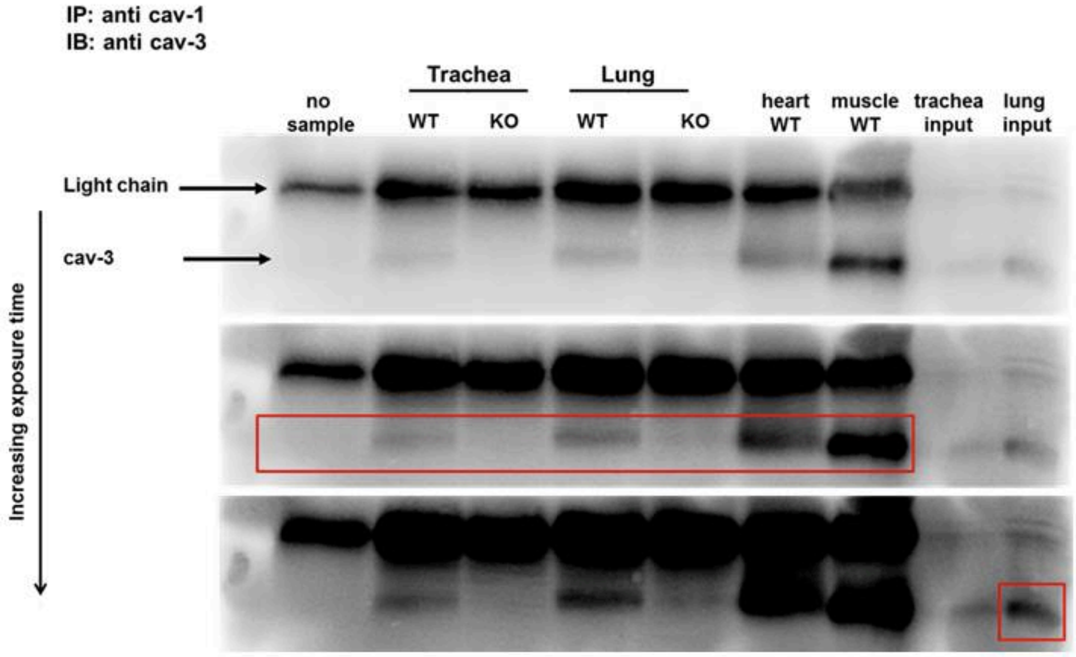

Fig. S3 Cav-3 interacts with cav-1 in cav-3$^{+/+}$ (WT) mice.

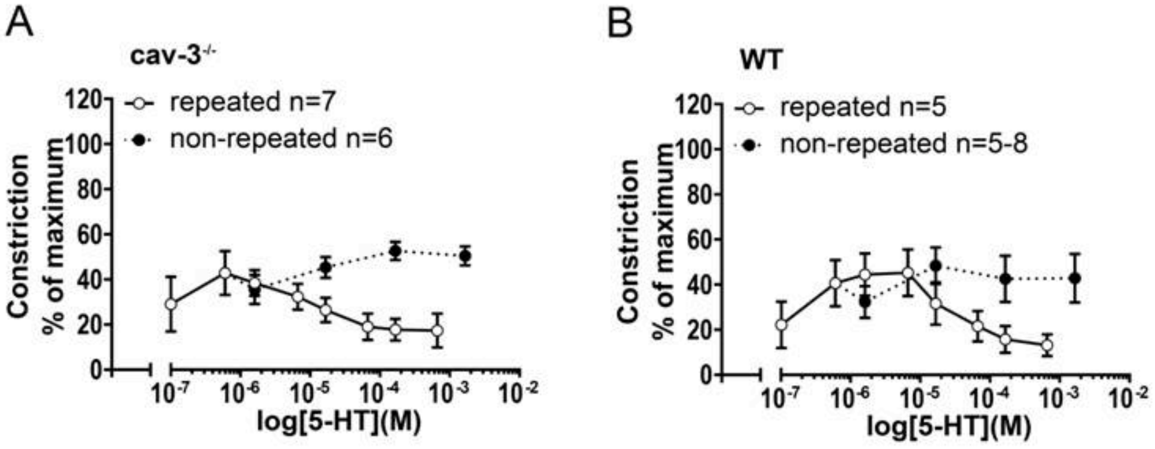

Fig. S4 Comparison of the response to 5-HT after repetitive stimulation with the response to 5-HT after non-repetitive stimulation.

Tab. S1 Oligonucleotide primers for cav-3 genotyping.

**Gene**	**Primer**	**Product length (bp) cav-3-*loxP***	**Product length (bp) cav-3$^{+/+}$**	**Product length (bp) cav-3$^{-/-}$**
**cav-3-8053-6F-5R**	fwd ttatgcgcaccatcagtc<br>rev cctaggtgtgctcatttg	368	323	-
**cav-3-8053-12F-5R**	fwd aaggaggcaatctgggctac<br>rev cctaggtgtgctcatttg	2000	1700	497
**Cre**	fwd gacaccaccagcaacacact<br>rev tccttgaagagcaggacgtg	390	390	390
